# Differential expression of ERCC-1 in the primary tumors and metastatic lymph nodes of patients with non-small cell lung cancer adenocarcinoma

**DOI:** 10.1007/s13277-012-0482-4

**Published:** 2012-08-14

**Authors:** Wen Zhang, Nannan Guo, Changhai Yu, Hongwei Wang, Yiming Zhang, Hui Xia, Jiangqi Yu, Jiangyang Lu

**Affiliations:** 1 Department of Cardiothoracic Surgery, The First Affiliated Hospital of General Hospital of the Chinese People’s Liberation Army, Fucheng Road 51, Beijing, 100048 People’s Republic of China; 2 Department of Pathology, The First Affiliated Hospital of General Hospital of the Chinese People’s Liberation Army, Fucheng Road 51, Beijing, 100048 People’s Republic of China

**Keywords:** Non-small cell lung cancer, Metastatic lymph node, Molecular markers, Serum markers

## Abstract

About 80 % of lung cancers are carcinomas that are classified histologically as non-small-cell lung carcinoma (NSCLC) and targeted chemotherapy of this cancer is currently based on sensitivity of the primary tumor to specific drugs. The purpose of this study was to compare the levels of four serum markers of cancer and the levels of six molecular markers which are possibly associated with drug selection in the primary tumors and metastatic lymph nodes of 39 consecutive NSCLC patients who were admitted to a single institution in China. Serum markers of cancer (neuron-specific enolase, carcinoembryonic antigen (CEA), cancer antigen 125, cytokeratin fragment 21-1) were measured by an automated electrochemiluminescence system and molecular markers (multidrug resistance protein 1, LDL receptor-related protein, ribonucleotide reductase M1, epidermal growth factor receptor, excision repair cross-complementing gene 1, and breast cancer 1) were measured by immunohistochemistry of the primary tumors and metastatic lymph nodes. The results indicate that the serum level of CEA was higher in NSCLC patients with adenocarcinoma relative to those with squamous cell carcinoma, but no significant differences in the other serum markers. Expression of excision repair cross-complementing gene 1 was significantly different in the primary tumors and metastatic sites of NSCLC patients with adenocarcinoma, but there were no other significant differences. This study provides an initial step toward the development of individualized chemotherapy of NSCLC based on measurement of molecular markers in the primary tumors and metastatic lymph nodes.

## Introduction

Lung cancer mortality in Western countries has declined due to a decrease in tobacco smoking in the past 20 years [1], but the incidence of cigarette smoking in China continues to increase and in the year 2005, an estimated 429,000 people died from lung cancer in China [2]. The most common treatments options for lung cancer are surgery, radiotherapy, and chemotherapy (which may be combined with radiotherapy and/or surgery).

There is great interest in development of targeted chemotherapeutic drugs that consider the unique molecular characteristics of each patient’s carcinoma [2]. For example, Brugger et al. [3] performed a large prospective biomarker study of patients with advanced non-small cell lung cancer (NSCLC) and reported that those with activating mutations of epidermal growth factor receptor (EGFR) benefit most from maintenance therapy with erlotinib. Kim et al. [4] studied chemorefractory NSCLC patients and found that patients with mutations in KRAS benefit most from sorafenib. However, the results of these and similar studies of the personalization of therapies for breast cancer [5], colorectal cancer [6, 7], and ovarian cancer [8] may be limited because the therapy is based on molecular markers in the primary tumor tissue, not in metastatic tissue.

Recent studies of Asian and Western patients have firmly established that lipoprotein receptor-related protein-1, ribonucleotide reductase M1 (RRM-1), EGFR, and excision repair cross-complementing gene 1 (ERCC-1) may be useful molecular markers to guide drug selection in patients with lung cancer [9–13]. Carcinoembryonic antigen (CEA) and carbohydrate antigen 125 (CA125) can also be used to guide diagnosis and treatment selection and as indicators of treatment efficacy for lung cancer [14, 15]. Cytokeratin-19 fragment (cytokeratin fragment 21-1 (CYFRA21-1)) is considered to be a sensitive marker for the diagnosis of lung squamous cell carcinoma [16, 17]. However, there have been no systemic studies on the possible differences in expression of these markers in primary and metastatic tissues.

After radical resection of primary lung tumor tissue, residual cancer cells are mainly located in the metastatic lymph nodes or other micro tumor tissues. Expression of biomarkers may be different in the primary tumors and residual metastatic lymph nodes, so these tissues may have different drug sensitivities. The different expression of these drug selection-associated genes at the primary tumor site and metastatic lymph nodes might account for the variable chemotherapeutic effectiveness of drugs which are selected mainly based on gene expression in the primary tumor.

In the present retrospective study, we examined the expression levels of these serum markers and compared the expression of molecular markers in the primary tumor tissues and metastatic lymph nodes of patients with NSCLC.

## Materials and methods

### Patient characteristics

This retrospective study enrolled all 39 NSCLC patients admitted to our hospital from September 2010 to October 2011 who underwent thoracic surgery for removal of primary lesions or removal of primary and metastatic lesions following pathological confirmation of lymph node metastasis. Each patient had a primary lung cancer lesion and at least one metastatic lymph node. In all included cases, lymph node metastasis was limited to the ipsilateral side lobe bronchus and the ipsilateral mediastinum (N1 and N2 stages) and could be completely removed by surgery. Post-operative chemotherapy consisted of paclitaxel + carboplatin, docetaxel + cisplatin, or gemcitabine + cisplatin. Some patients were given targeted therapy with gefitinib. NSCLC staging was based on the TNM Classification of Malignant Tumors, Seventh Edition [18].

### Specimen processing and staining

During thoracic surgery, the primary lesions and lymph nodes were resected and specimens were stored in 10 % neutral formalin for 24 h. Samples were sectioned at a thickness of 2 mm, tissue was dehydrated, embedded in paraffin, cut into 5 μm sections, and dried overnight at 37 °C.

For hematoxylin-eosin staining, tissue slides were immersed in a jar with fresh 15 % H_2_0_2_ for 10 min, washed thoroughly in tap water, stained in Harris’s haematoxylin for 15 min, and then washed again in tap water. A solution of 1 % aqueous eosin Y with two drops of concentrated acetic acid was added to the jar for 5 min for counterstaining. Tissue slides were then washed thoroughly, air dried, and cover-slipped with Permount medium (Fischer, USA) for microscopic examination.

Immunohistochemical staining of the six molecular markers (multidrug resistance protein 1 (MDR-1), LRP, RRM-1, EGFR, ERCC-1, and breast cancer 1 (BRCA-1)) was performed as described by Selvaggi et al. [19]. All reagents and antibodies were purchased from Beijing Golden Bridge Biotechnology Co., Ltd. and the dilutions and applications of primary antibodies were according to the manufacturer’s instructions. The secondary antibody was horseradish peroxidase and the chromogenic substrate was 3,3′-diaminobenzidine.

### Immunohistochemical analysis

Samples were viewed at high magnification (×400) and eight fields of each sample were visualized for semiquantitative analysis. The total staining score was based on a system previously described by Fromowitz et al. [20]. In particular, each field was scored as “0” (no staining), “1” (light yellow staining), “2” (light brown staining), or “3” (dark brown staining). The overall percentage of positive staining per field was scored as “0” (≤5 % staining), “1” (6–25 % staining), “2” (26–50 % staining), “3” (51–75 % staining), or “4” (>75 % staining). The final score was simply the sum of these two individual scores, and was “−” (0–1 points), “+” (2–3 points), “++” (4–5 points), or “+++” (6–7 points).

### Measurement of serum tumor markers

Serum samples were taken from all patients before surgery, centrifuged at 2,000×*g* for 25 min, and stored at −20 °C prior to analysis. The COBAS 6000 automatic electrochemiluminescence immunoassay analyzer (Roche) was used to measure levels of neuron-specific enolase (NSE), CEA, CA125, CYFRA21-1. All reagents were from Roche. The normal ranges of these markers are: NSE, 0–15 μg/mL; CEA, 0–3.4 ng/mL; CA125, 0–35 U/mL; and CY21-1, 0–3.3 ng/mL.

### Statistical analysis

The Mann–Whitney *U* test was used to compare the expression of tumor markers and Fisher’s exact test was used to compare categorical variables. Results are given as median (interquartile range) for tumor markers and as number (number) for categorical data. The Wilcoxon signed ranks test was used to compare differences in the expression of molecular markers in primary lesions and metastatic lymph nodes. Spearman’s correlation coefficient was used to determine the relationship between ERCC-1 and CEA levels. All statistical assessments were two-sided and evaluated at the 0.05 level of significance. Bonferroni correction was used for multiple comparisons. Statistical analyses were performed using SPSS 15.0 statistics software (SPSS Inc, Chicago, IL, USA).

## Results

We retrospectively reviewed the records of all NSCLC cancer patients who underwent thoracic surgery in our hospital from September 2010 to October 2011 (Table 1). Ultimately, we examined the records of 39 patients with primary lung cancer lesions and at least one metastatic lymph node, all of whom underwent surgery for removal of the primary and metastatic lesions. The patients included 30 men and nine women and the mean age was 59.54 ± 10.41 years (range, 38–78 years). A total of 24 patients had squamous cell lung carcinoma and 15 had adenocarcinoma. Twenty-nine patients (74.4 %) were tobacco smokers.

**Table 1:** Demographic and clinical characteristics of enrolled NSCLC patients (*n* = 39)

Variable	
Age, years ± standard deviation	59.54 ± 10.41
Gender, *n* (%)	
Male	30 (76.9 %)
Female	9 (23.1 %)
Subtype of lung cancer, *n* (%)	
Squamous cell carcinoma lung cancer	24 (61.5 %)
Adenocarcinoma lung cancer	15 (38.5 %)
Smoker, *n* (%)	29 (74.4 %)

First, we compared the expression of six molecular biomarkers (MDR-1, LRP, RRM-1, EGFR, ERCC-1, and BRCA-1) in the primary lung carcinoma and the metastatic lymph nodes of patients with the two subtypes of NSCLC (Table 2). The results indicate no significant differences in the scores for expression of these biomarkers in patients with these different NSCLC subtypes.

**Table 2:** Differences in biomarker expression scores (see “Materials and methods”) of primary lesions and metastatic lymph nodes of patients with different subtypes of NSCLC

	Squamous cell (*n* = 24)	Adenocarcinoma (*n* = 15)	*P* value
MDR-1			
Decreased	3 (12.5 %)	1 (6.7 %)	1.000
No change	18 (75.0 %)	12 (80.0 %)	
Increased	3 (12.5 %)	2 (13.3 %)	
LRP			
Decreased	2 (8.3 %)	1 (6.7 %)	1.000
No change	15 (62.5 %)	10 (66.7 %)	
Increased	7 (29.2 %)	4 (26.7 %)	
RRM-1			
Decreased	5 (20.8 %)	6 (40.0 %)	0.200
No change	17 (70.8 %)	6 (40.0 %)	
Increased	2 (8.3 %)	3 (20.0 %)	
EGFR			
Decreased	5 (20.8 %)	4 (26.7 %)	0.907
No change	14 (58.3 %)	8 (53.3 %)	
Increased	5 (20.8 %)	3 (20.0 %)	
ERCC-1			
Decreased	6 (25.0 %)	6 (40.0 %)	0.389
No change	16 (66.7 %)	9 (60.0 %)	
Increased	2 (8.3 %)	0 (0.0 %)	
BRCA-1			
Decreased	1 (4.2 %)	3 (20.0 %)	0.062
No change	21 (87.5 %)	8 (53.3 %)	
Increased	2 (8.3 %)	4 (26.7 %)	

*P* values are from Fisher’s exact test

**Fig. 1:**
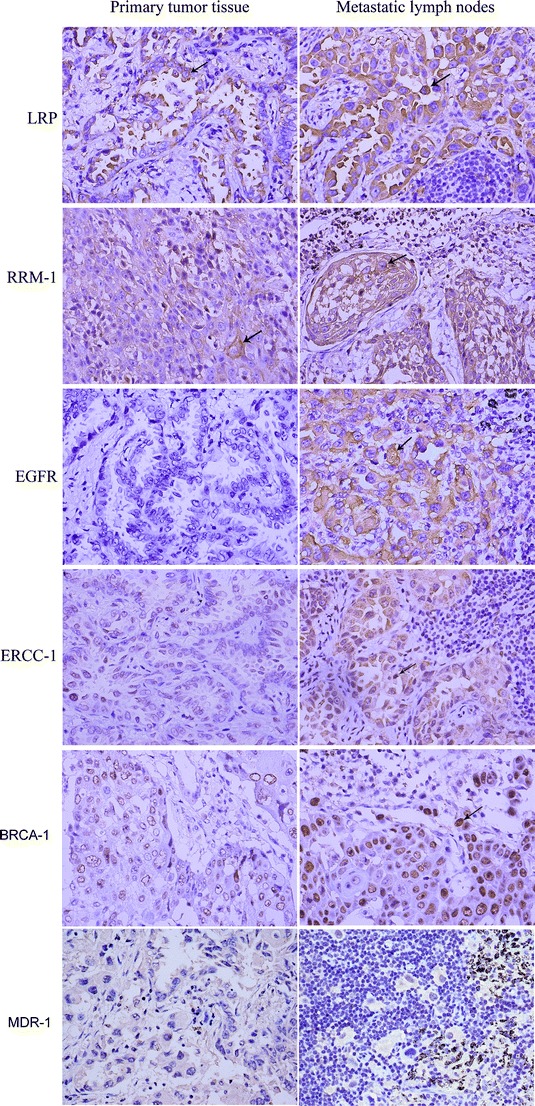
Representative immunohistochemical staining results for LRP, RRM-1, EGFR, and ERCC-1, BRCA-1, and MDR-1 in a primary tumor (adenocarcinoma) and a metastatic lymph node (squamous cell carcinoma), ×400. *Arrows* indicate strong positive staining in the cytoplasma and the nucleus

Next, we compared the expression scores of the six molecular markers in the primary lesions and metastatic lymph nodes of all 39 patients. Figure 1 shows representative immunohistochemical staining results for LRP, RRM-1, EGFR, ERCC-1, BRCA-1, and MDR-1 in primary lesions and metastatic lymph nodes. RRM-1, LRP, and MDR-1 were positively stained in cytoplasm of tumor cells in both primary lesion and metastatic lymph node. EGFR was positively stained in cytoplasmic membrane in metastatic lymph node but not in primary lesion. ERCC-1 was positively stained in the nucleus in metastatic lymph node but not in primary lesion. BRCA-1 was positively stained the cytoplasm in metastatic lymph node but not in primary lesion. Analysis of these results indicates that ERCC-1 expression was significantly different in the primary lesions and metastatic lymph nodes of patients with adenocarcinoma (Table 3). There were no other significant differences in the expression of markers in the primary tumors and metastatic lymph nodes.

**Table 3:** Expression of molecular markers in the primary lesions and metastatic lymph nodes of patients with different subtypes of NSCLC

	Squamous cell (*n* = 24)			Adenocarcinoma (*n* = 15)		
	Primary lesion (%)	Metastatic lymph nodes (%)	*P* value	Primary lesion (%)	Metastatic lymph nodes (%)	*P* value
MDR-1						
−	20 (83.3)	20 (83.3)	0.739	13 (86.7)	14 (93.3)	0.564
±	3 (12.5)	3 (12.5)		0 (0.0)	0 (0.0)	
+	0 (0.0)	1 (4.2)		2 (13.3)	1 (6.7)	
++	1 (4.2)	0 (0.0)		0 (0.0)	0 (0.0)	
+++	0 (0.0)	0 (0.0)		0 (0.0)	0 (0.0)	
LRP						
−	13 (54.2)	15 (62.5)	0.140	0 (0.0)	1 (6.7)	0.480
±	0 (0.0)	1 (4.2)		0 (0.0)	0 (0.0)	
+	7 (29.2)	6 (25.0)		4 (26.7)	4 (26.7)	
++	4 (16.7)	2 (8.3)		4 (26.4)	3 (20.0)	
+++	0 (0.0)	0 (0.0)		7 (46.7)	0 (0.0)	
RRM-1						
−	5 (20.8)	4 (16.7)	0.206	3 (20.0)	3 (20.0)	0.151
±	0 (0.0)	0 (0.0)		1 (6.7)	0 (0.0)	
+	9 (37.5)	9 (37.5)		3 (20.0)	2 (13.3)	
++	6 (25.0)	5 (20.8)		5 (33.3)	5 (33.3)	
+++	4 (16.7)	6 (25.0)		3 (20.0)	5 (33.3)	
EGFR						
−	2 (8.3)	3 (12.5)	0.782	4 (26.7)	2 (13.3)	0.380
±	1 (4.2)	0 (0.0)		0 (0.0)	1 (6.7)	
+	3 (12.5)	4 (16.7)		2 (13.3)	2 (13.3)	
++	14 (58.3)	12 (50.0)		6 (40.0)	7 (46.7)	
+++	4 (16.7)	5 (20.8)		3 (20.0)	3 (20.0)	
ERCC-1						
−	18 (75.0)	17 (70.8)	0.201	11 (73.3)	8 (53.3)	0.026*
±	1 (4.2)	2 (8.3)		2 (13.3)	1 (6.7)	
+	3 (12.5)	0 (0.0)		2 (13.3)	4 (26.7)	
++	2 (8.3)	3 (12.5)		0 (0.0)	2 (13.3)	
+++	0 (0.0)	2 (8.3)		0 (0.0)	0 (0.0)	
BRCA-1						
−	17 (70.8)	19 (79.2)	1.000	8 (53.3)	8 (53.3)	0.260
±	2 (8.3)	1 (4.2)		0 (0.0)	4 (26.7)	
+	5 (20.8)	3 (12.5)		7 (46.7)	3 (20.0)	
++	0 (0.0)	0 (0.0)		0 (0.0)	0 (0.0)	
+++	0 (0.0)	1 (4.2)		0 (0.0)	0 (0.0)	

*P* values are from Wilcoxon signed ranks test. The scoring system (−, ±, +, ++, +++) is described in the “Materials and Methods” section

*P < 0.05, significant difference

**Fig. 2:**
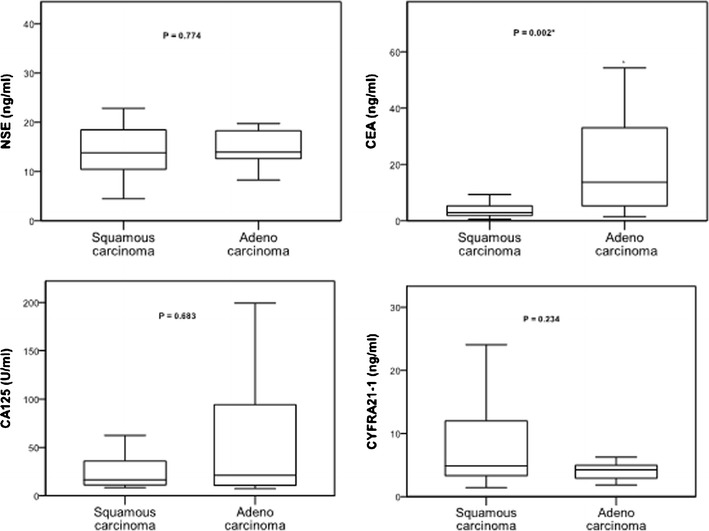
Expression of serum tumor markers (NSE, CEA, CA 125, and CYFRA 21-1) in patients with different subtypes of NSCLC

**Fig. 3:**
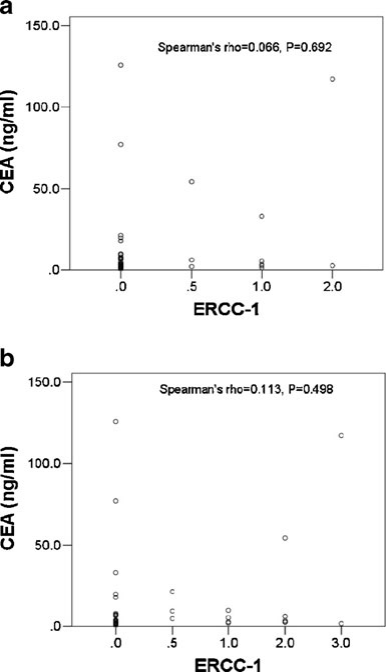
The correlation between ERCC-1 and CEA levels in the primary tumor tissue (**a**) and the metatstatic lymph nodes (**b**)

Finally, we examined the levels of four serum markers of cancer (NSE, CEA, CA 125, and CYFRA 21-1) in the same 39 patients (Fig. 2). The results indicate no significant differences in NSE, cancer antigen 125 (CA-125), and CYFA21-1, but significantly higher expression of CEA in patients with adenocarcinoma lung cancer relative to those with squamous cell lung carcinoma (*p* = 0.002). In addition, the correlations between ERCC-1and CEA levels in the primary lesions (Fig. 3a, *P* = 0.692) and metastatic lymph nodes (Fig. 3b, *P* = 0.498) were not statistically significant.

## Discussion

We studied 39 consecutive NSCLC patients and measured the expression of six molecular markers (MDR-1, LRP, RRM-1, EGFR, ERCC-1, BRCA-1) in their primary tumors and metastatic lymph nodes and four well-known serum markers for cancer (NSE, CEA, CA-125, CYFRA 21-1). Our results indicate that ERCC had significantly different expression in the primary tumors and metastatic lymph nodes of patients with adenocarcinoma. However, there were no other significant differences in the expression of the markers in the primary tumors and metastatic lymph nodes. Our measurements of serum markers indicated that serum CEA level was significantly higher in patients with adenocarcinoma rather than squamous cell carcinoma, but there were no other differences in expression of the serum markers that we measured. These results provide an initial step toward the development of lung cancer therapy that is based on measurement of the expression of biomarkers in the primary tumor tissue, metastatic lymph nodes, and serum.

Individualized treatment of cancer is believed to have great promise and many clinical and experimental studies have used tumor-specific molecular markers to identify differences in patients in order to better estimate prognosis and select treatments [21]. This motivated our comparison of the expression of six molecular markers in the primary tumors and metastatic lymph nodes of patients with NSCLC.

We carefully selected the markers that we studied. LRP is the major vault protein whose elevated expression is associated with poor response to chemotherapy [22, 23]. Rybarova et al. [24] reported that LRP expression was significantly greater in patients with NSCLC than in those with SCLC, which is in line with the general clinical finding that untreated SCLC is more chemosensitive than untreated NSCLC. Our results indicated no significant differences in LRP expression in primary and metastatic lesions of patients with NSCLC. Ribonucleotide reductase is a rate-limiting enzyme for the synthesis of DNA and has two subunits, RRM-1 and RRM-2. The RRM-1 gene is a target of numerous chemotherapeutic agents [25]. Previous clinical studies have shown that lung cancer patients with low levels of RRM-1 mRNA are more sensitive to gemcitabine and have longer survival times [26–28] and the recent US National Comprehensive Cancer Treatment Access Coalition (NCCN) NSCLC treatment guidelines recommend measurement of RRM-1 expression before implementation of gemcitabine therapy in NSCLC patients [29]. Our results indicated no significant differences in RRM-1 expression in primary and metastatic lesions of patients with NSCLC. EGFR is a receptor tyrosine kinase involved in activation of transcription factors that regulate gene transcription, cell migration, adhesion, differentiation, and apoptosis [30]. EGFR-positive lung cancer patients are more responsive to Iressa and Tarceva [31], which are classified as EGFR tyrosine kinase inhibitors. Our results indicated no significant differences in EGFR expression in primary and metastatic lesions of patients with NSCLC. ERCC-1 is a key enzyme in nucleotide excision repair and mutations in this gene appear to play a role in cancer pathogenesis [13]. Previous clinical studies have demonstrated that downregulation of ERCC-1 in NSCLC patients is associated with increased sensitivity to platinum-based chemotherapy [32, 33]. The recent NCCN NSCLC treatment guidelines recommend measurement of ERCC-1 before implementation of platimum-based chemotherapy in NSCLC patients [29]. Interestingly, we found significantly different expression of ERCC-1 in the metastatic and primary lesions of NSCLC patients with adenocarcinoma. Das et al. [34] recently reported that their use of a novel circulating tumor cell (CTC) blood test to measure ERCC-1 expression in CTCs in patients with metastatic NSCLC. They found that low expression of ERCC1 on CTCs correlated with progression-free survival. These findings provide general support for our finding of the importance of measuring markers outside the primary tumor.

Previous studies indicate that genetic testing should be performed on lung cancer patients and that the treatment should be customized according to the results. Taken together, our results indicate that MDR-1, LRP, RRM-1, EGFR, and BRCA-1 levels were similar in the primary and metastatic lesions of all NSCLC patients. This was not surprising, because these lesions ultimately have the same source. However, ERCC-1 levels were significantly different in the primary and metastatic lesions. At present, the cause and clinical significance of this difference is uncertain. It is possible that the cells of the primary lung tumor which become metastatic are genetically unique from the bulk of the primary tumor cells [35]. We suggest that future studies measure ERCC-1 in primary and metastatic lesions and determine the association of altered ERCC-1 levels with patient prognosis and responsiveness to different chemotherapy regimens.

Our measurement of serum markers of cancer indicated that NSE, CA-125, and CYA 21-1 levels were similar in NSCLC patients with squamous cell carcinoma and adenocarcinoma. However, CEA levels were significantly higher in NSCLC patients with adenocarcinoma. Again, we suggest that future studies measure serum CEA levels in NSCLC patients and determine the association of CEA 1 levels with patient prognosis and responsiveness to different chemotherapy regimens. But we did not find any relationship between ERCC-1 and CEA levels in the primary tumor tissues or in the metastatic lymph nodes. ERCC-1 and CEA might be associated with lung cancer development and its metastasis through different mechanism. How these two molecules involved in the lung cancer development and metastasis should be further investigated in future study.

Our study had several limitations that should be noted. First, our sample size was relatively small, limiting the statistical power of our results. Second, this study was performed at a single institution, so the results should not be generalized to other institutions. Third, this was a retrospective study, so there may have been significant selection bias.

At present, the onset and pathogenesis of NSCLC are not completely understood and predictions of prognosis are not very reliable. The use of molecular markers to guide treatment of this cancer is currently in the initial stages. Our results suggest that expression of ERCC-1 was significantly different in primary tumors and metastatic sites of NSCLC patients with adenocarcinoma and that the serum level of CEA was significantly higher in NSCLC patients with adenocarcinoma. Large-scale multi-institutional prospective studies are needed to validate these findings before we can make definitive suggestions about the use of individualized treatment based on measurement of these biomarkers in primary tumors and metastatic lymph nodes.
